# Clinician insights into pediatric temporary feeding tube management: Unseen barriers, unclear roles revealed from a prospective mixed methods study

**DOI:** 10.1002/ncp.70093

**Published:** 2026-02-11

**Authors:** Claire Reilly, Rebecca Packer, Jeanne Marshall, Nikhil Thapar, Jasmine Foley

**Affiliations:** ^1^ School of Health & Rehabilitation Sciences, The University of Queensland Brisbane Australia; ^2^ Queensland Children's Hospital, Children's Health Queensland Hospital and Health Service South Brisbane Australia; ^3^ Department of Gastroenterology, Hepatology and Liver Transplant Queensland Children's Hospital (QCH) Brisbane Australia; ^4^ Faculty of Health School of Exercise & Nutrition Sciences, Queensland University of Technology Brisbane Australia; ^5^ School of Medicine, The University of Queensland Brisbane Australia

**Keywords:** children, clinicians, temporary tube feeding

## Abstract

**Background:**

Temporary feeding tubes are common in pediatric healthcare, but research on understanding how clinicians manage their use and understand the impact on families is limited. Existing research often overlooks clinician perspectives despite tensions between clinical priorities and family needs. This study aimed to understand clinicians' insights into temporary pediatric feeding tube management, focusing on communication, decision‐making, and family‐centered practice.

**Methods:**

Using a mixed‐methods design, this study integrated quantitative survey data from multidisciplinary clinicians at a leading teaching hospital with qualitative insights from in‐depth interviews. Analysis involved descriptive statistics for survey data and reflexive thematic analysis for interviews, as well as a comprehensive synthesis of data.

**Results:**

Survey data from 112 multidisciplinary clinicians (54% response rate) and interviews with 12 clinicians revealed tensions between clinical intentions and available resources. Qualitative themes highlighted role ambiguity across disciplines, multilevel systemic barriers, an expanding awareness of comprehensive family burdens, and how clinician perceptions can potentially shape family experiences with temporary feeding tubes. These findings underscore the complexities of navigating temporary feeding tube management within the existing healthcare system.

**Conclusion:**

This study describes how clinicians navigate temporary feeding tube management within complex healthcare environments, drawing on collaborative expertise while working with limited standardized guidance. Findings reveal evidence‐practice gaps, role ambiguities, and system constraints that limit clinicians' ability to provide family‐centered care. Addressing these challenges necessitates structured tools, training, and systemic reform to better support clinicians in delivering family‐centered management.

## INTRODUCTION

Pediatric temporary feeding tube management is widely used in healthcare worldwide to provide short‐term nutrition support for children unable to meet their nutrition requirements.^1^, ^2^, ^3^ Temporary feeding tubes may be required by up to 25% of hospitalized children,^3^ with research showing that 17% are discharged home with these tubes in place.^4^ Despite widespread use of temporary feeding tubes, there is a lack of standardized, evidence‐based practice guidelines to clinical implementation and management regarding their use.^5^, ^6^, ^7^, ^8^, ^9^, ^10^ Preliminary studies have identified that in the absence of formal guidelines, clinicians often draw on institutional culture and informal hierarchies,^11^, ^12^ which can create widespread variation in how temporary tube feeding is approached and managed.^13^, ^14^, ^15^

The literature demonstrates considerable clinical variations across multiple aspects of temporary tube feeding management, including consensus on what is considered a temporary tube, the selection of feeding tube, and criteria for tube removal. Firstly, variability in the definition and duration of temporary feeding tubes creates a fundamental challenge, specifically, how long constitutes as temporary remains open to individual clinical interpretation, with durations varying significantly across practice settings.^5^, ^8^, ^10^, ^16^ Secondly, selecting a type of feeding tube has been demonstrated to be predominantly determined by clinician familiarity,^17^ procedural expertise,^18^ and local protocols,^5^ rather than what is regarded as optimal for clinical care. Without standardized criteria, clinicians may approach tube selection differently, creating wide variations in management despite similar clinical needs and what may be deemed optimal for clinical care. Similarly, research has shown that fewer than half of hospital guidelines internationally include tube removal criteria.^6^ Collectively, these interconnected variations indicate that clinicians are relying on limited evidence, clinical experience, and institutional historical practice to guide temporary tube insertion, management, and removal.

Although these practice variations highlight the reliance on limited evidence for temporary tube feeding management, the existing body of research has predominately focused on the management of children with long‐term (eg, gastrostomy) feeding tubes.^19^, ^20^, ^21^, ^22^ This research shows us that standardized, evidence‐based guidelines are both feasible and beneficial for feeding tube management. However, these systematic reviews, position papers, and recommendations are specific to long‐term feeding tubes, creating an urgent need to develop equivalent standardized guidelines for temporary tube feeding management. Several research studies regarding the management of temporary feeding tubes do exist, but these studies predominantly originate from neonatal intensive care units (NICUs) that have highly structured guidelines and protocols within this complex clinical setting.^23^, ^24^, ^25^, ^26^, ^27^, ^28^, ^29^, ^30^ These NICU‐based findings have informed a systematic review recommendation to apply the findings to broader temporary tube feeding applications outside the NICU,^31^ yet such recommendations fail to consider important clinical drivers and differences among settings, such as resource disparities between a NICU environment and other pediatric settings.

Existing research in a broader pediatric setting has reported training deficits and knowledge gaps regarding temporary tube placement and removal.^9^, ^12^, ^15^, ^32^ In addition to this, studies have shown that up to 50% of clinicians report uncertainty about tube weaning timing,^33^ limited awareness of guidelines, and inconsistent implementation even when local guidelines exist.^34^, ^35^ To move toward evidence‐based standards of care, we must first understand the drivers that influence temporary feeding tube management in clinical practice. Therefore, the aim of the current study was to explore clinicians' insights into temporary pediatric feeding tube management, examining their communication approaches, decision‐making processes, and family‐centered practice perceptions.

## METHODS

### Study design and setting

This mixed‐methods design study integrated quantitative survey data with qualitative interviews at a single site, 377‐bed public quaternary children's hospital in Brisbane, Australia. Survey data were collected from August to December 2023, and interviews were conducted from April to November 2024. Ethical approval was granted from the Children's Health Queensland Hospital and Health Service, Human Research Ethics Committee (Reference number HREC/23/QCHQ/94925), and The University of Queensland Human Research Ethics & Integrity Committee (Reference number: 2023/HE000816) before the commencement of the study. Both committees serve as Institutional Review Boards (IRBs) under Australian regulations. Electronic informed consent was obtained from all clinician participants via the online platform prior to survey participation. Clinicians could opt‐in to participate in an optional interview via this same survey.

### Participation and recruitment

Clinicians eligible to participate in the survey included nurse specialists, midwives, medical practitioners, occupational therapists, speech pathologists, and dietitians involved in temporary tube feeding care. Purposive sampling captured diverse perspectives across disciplines from clinicians who had been involved in caring for children discharged home with temporary feeding tubes. Recruitment occurred over 4 months via email invitations with two reminder emails. Participation was voluntary, with informed consent obtained before survey completion. The survey achieved a 54% response rate (*n* = 112). Survey participants could indicate a willingness to be contacted for a follow‐up interview, and those who agreed were later contacted and invited to participate in semistructured interviews.

### Terminology used in this study

Clinicians is an umbrella term that includes physicians, nurses, midwives, and allied health clinicians involved in a child's care. Medical team refers to the physicians primarily involved in a child's care (eg, respiratory team). Medical practitioner refers to physicians. Allied health clinicians refer to speech‐language pathologists, dietitians, and occupational therapists. Allied health team refers to these allied health clinicians involved in a child's tube feeding care. Multidisciplinary team refers to clinicians from more than one discipline who are involved in a child's care. In this study, multidisciplinary team typically involved the medical team with one or more of dietetics, speech‐language pathology, occupational therapy, and nursing. The term multidisciplinary meeting is used only when a formal meeting was described.

### Data collection

#### Survey development and administration

The survey was developed based on a comprehensive review of the literature, established clinician survey research principles,^36^ and the clinical expertise of the research team. Initial concepts were refined through consultation with six medical teams and the dietetics team at the participating hospital, then pilot tested with three experienced clinicians (one allied health clinician and two medical practitioners) who assessed clarity, feasibility, usability, and content. Clinicians emphasized brevity and the preference for closed‐ended multiple‐choice questions. All suggestions were incorporated by consensus, ensuring the survey was both clinically relevant to the research question and feasible for busy hospital‐based clinicians. The final survey comprised mainly closed‐ended questions: five‐point Likert‐type items capturing agreement (strongly disagree to strongly agree) and frequency (never to always), plus multiple‐choice questions. Open‐text response fields were also provided in several items through an “Other (please specify)” option, allowing participants to elaborate on or add responses not listed. These appeared in questions about information sources, decision‐making approaches, and involvement in care decisions. A single, entirely open‐ended question, “Do you have any final comments you would like to add?,” was included at the end of the survey to enable broader, unsolicited reflections. The survey also addressed some patient‐specific items. These data are reported separately^37^ as they reflect distinct research aims outside the scope of this study. The final survey was administered online via Qualtrics,^38^ a secure web‐based platform. To minimize social desirability bias in the hospital setting, in which the principal investigator was also a clinician, the survey was anonymous.^39^ The average completion time was approximately 5 min. The survey is provided in File S1.

#### Interview development and process

To ensure that clinician interview questions addressed real‐world family challenges, they were informed by multiple sources, including insights from parent diaries/interviews conducted in another component of this study^40^, ^41^ and existing literature. To assess clarity, feasibility, and relevance, cognitive interviewing was conducted with three experienced clinicians external to the participating hospital, following the framework outlined by Izumi et al.^42^ This process allowed for refinement of question phrasing, structure, and flow, ensuring they were clear, nonleading, and reflective of the study's objectives. The interview guide is provided in File S2. To minimize bias, trained external interviewers R. P. and J. F. facilitated all interviews.

### Data analysis

#### Quantitative analysis

Descriptive statistical analysis was performed using Microsoft Excel (Version 2502). For Likert scale survey questions, response frequencies and weighted averages were calculated by assigning numerical values to response levels. Computed means were categorized into frequency levels for clearer interpretation, with means of 1.0–2.5 classified as “low,” 2.6–3.5 as “moderate,” and 3.6–5.0 as “high” based on the five‐point Likert scale range.^43^ For open‐ended questions, *n* indicates the number of respondents who provided open‐ended responses. Open‐ended survey responses were analyzed using basic content analysis following the methods of Graneheim and Lundman^44^ to identify recurring patterns and categorize similar responses into common themes. Since these responses were submitted via “Other (please specify)” options in survey items, participants typically provided brief, one‐line answers rather than elaborated reflections. This limited the depth of interpretation but offered useful insight into additional perspectives not captured by the fixed‐response options.

#### Qualitative interview analysis

Interview data were analyzed using reflexive thematic analysis following Braun and Clarke.^45^ This process involved familiarization with the transcripts, generating initial codes, developing and reviewing themes, and defining and naming themes. The primary author initially coded interview transcripts and participated in the iterative refinement of codes into meaningful themes alongside author J. F. through team discussions. Themes were systematically checked against the original data to ensure they accurately represented participants' accounts, aligned with the study aims. Disagreements were resolved through discussion and checking with other members of the research team until consensus was reached. NVivo software (version 14) was used to manage and organize the qualitative data.

#### Positionality

The primary investigator is a pediatric dietitian with nearly two decades of clinical experience in temporary tube feeding management, currently completing a doctorate degree focused on family experiences. This background influenced data interpretation by focusing on understanding the relationship between clinical practice and family needs. To acknowledge the influence of the researcher's experience on the interpretation of the data, positioning was discussed in meetings throughout analysis, and written reflections were maintained for transparency.

## RESULTS

**Figure 1:**
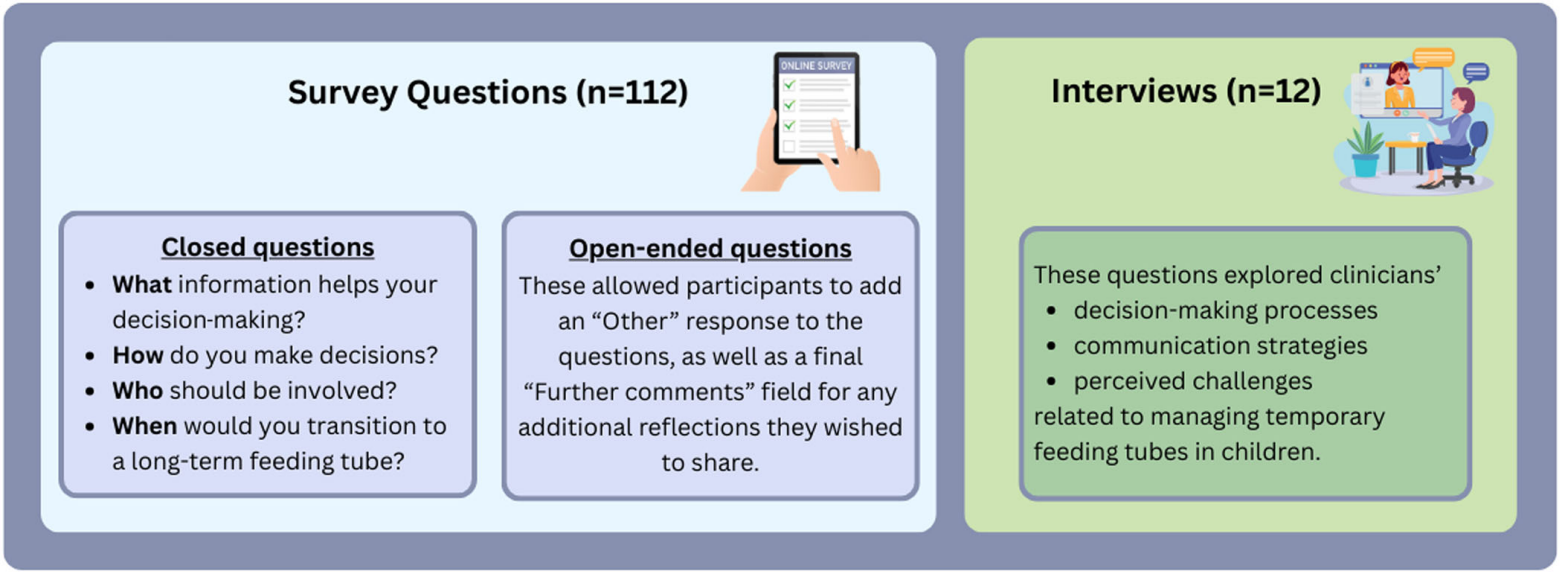
Data collection methods overview. Mixed‐methods approach, including survey questions (*n* = 112) with closed and open‐ended components, and semistructured interviews (*n* = 12) exploring perspectives of temporary feeding tube management.

Results are presented in three sections: (1) closed‐question survey responses, (2) open‐ended survey responses, and (3) clinician interviews, as described in Figure 1.

### Closed‐question survey question responses (quantitative)

The survey was completed by 112 clinicians (54% response rate), including a combination of medical practitioners, speech pathologists, occupational therapists, dietitians, and nurses. Individual respondent characteristics could not be specified because of survey anonymity.

The quantitative portion of the survey explored three key areas of clinical practice, including (1) information sources used in decision‐making, (2) decision‐making approaches used, and (3) recommended timelines for transitioning children from temporary to long‐term feeding tubes, described in Table 1.

**Table 1:** Clinician survey results.

Survey items	Response options	*n*	Never, %	Sometimes, %	About half the time, %	Most of the time, %	Always, %	Computed mean	Computed SD	Frequency of use
What information helps your decision‐making, and how often do you use it?	Hospital guidelines/policies	111	12.6	42.3	12.6	23.4	9.0	2.7	1.2	Moderate
	Online clinical database	106	49.1	36.8	10.4	2.8	0.9	1.7	0.8	Low
	Online search	108	31.5	48.1	10.2	9.3	0.9	2.0	0.9	Low
	Medical team	111	0.0	4.5	6.3	46.8	42.3	4.3	0.7	High
	Dietitians	111	0.0	3.6	5.4	46.8	44.1	4.3	0.7	High
	Research journal articles	110	30.0	54.5	10.0	5.5	0.0	1.9	0.7	Low
	Workshops/conferences	109	35.8	45.9	11.0	6.4	0.9	1.9	0.9	Low
								2.7^a^		
How do you typically make decisions about a child's temporary feeding tube?	Discuss with medical team	112	0.0	3.6	25.0	1.8	69.6	4.4	1.0	High
	Discuss with allied health	112	0.0	0.9	4.5	38.4	56.2	4.5	0.6	High
	Discuss with the family	112	0.0	0.9	0.9	19.6	78.6	4.8	0.5	High
	A multidisciplinary meeting	112	7.1	48.2	24.1	15.2	5.4	2.6	1.0	Low
								4.1^a^		
Who should be involved in the decision‐making about the child's temporary feeding tube?	Treating medical team	111	0.9	0.0	0.0	92.8	6.3	4.0	0.4	Moderate
	Dietitians	111	0.9	0.0	0.9	9.9	88.3	4.8	0.5	High
	Parents/caregivers	110	0.9	0.0	1.8	7.3	90.0	4.9	0.5	High
	Feeding therapists	110	0.9	0.0	4.5	23.6	70.9	4.6	0.7	High
								4.6^a^		

*Note*: Means and standard deviations are based on weighted Likert scale responses. Frequency of use categories are defined as follows, based on computed means: low, 1.0–2.5; moderate, 2.6–3.5; and high, 3.6–5.0. Medical practitioners are physicians only. Allied health clinicians are dietitians, speech‐language pathologists, and occupational therapists. Clinicians include physicians, nurses, midwives, and allied health clinicians. Multidisciplinary team indicates more than one discipline.

^a^ Weighted average.

#### Information sources

Clinicians reported frequent reliance on their colleagues: dietitians 44.1% (*n* = 49/111) and the treating medical team 42.3% (*n* = 47/111) were “always” used. Hospital guidelines were “always” used by 9% (*n* = 10/111). Other sources were rarely “always” used: research journals 0%, clinical databases 0.9% (*n* = 1/106), online searches 0.9% (*n* = 1/108), and workshops/conferences 0.9% (*n* = 1/109).

#### Decision‐making approaches

Ad hoc meetings were common. Decisions were “always” discussed with families (78.6%, *n* = 88/112), medical teams (69.6%, *n* = 78/112), and allied health (56.2%, *n* = 63/112). Formal multidisciplinary meetings were rarely “always” used (5.4%, *n* = 6/112).

#### Timing of transition from temporary feeding tube to long‐term feeding tubes

Recommendations varied: 27.8% (*n* = 22/79) advised transitioning at 4–6 months, 43% (*n* = 34/79) at 7–12 months, and 29.1% (*n* = 23/79) at 13–18 months.

#### Who should be involved in decision‐making

A strong consensus was for involving parents/caregivers (90.0%, *n* = 99/110) “always”, dietitians 88.3% (*n* = 98/111) “always,” and feeding therapists 70.9% (*n* = 78/110) “always.” For the treating medical team, most selected “most of the time” (92.8%, *n* = 103/111) rather than “always” (6.3%, *n* = 7/111).

### Open‐ended survey question responses (qualitative insights)

Responses to the open‐ended survey items (provided as open‐text responses) varied widely, with the number of responses per item ranging from 1 to 31.

#### Who should be involved in decision‐making (*n* = 11)

In addition to the listed survey options, 11 clinicians provided open‐text responses identifying additional roles they believed should be involved in decision‐making. One of these responses came from a different survey item asking (“how do you typically make decisions…”), but the answer aligned with this question and was included. Most responses (*n* = 8) emphasized nursing roles, such as ward nurses, clinical nurse consultants, lactation consultants, and Hospital‐in‐the‐Home nurses, as important yet often excluded decision‐makers. Fewer responses highlighted other stakeholders, including psychologists or mental health clinicians, community services, or the child themselves if age appropriate. One respondent noted that the appropriate decision‐makers may vary depending on the acuity and chronicity of the child's condition.

#### Transitioning to a long‐term feeding tube (*n* = 31)

Thirty‐one clinicians provided open‐text responses describing their approaches to transitioning a child from a temporary to a long‐term feeding tube. Reported timeframes varied widely, from 1 month to 2 years. Most referenced clinical indicators, such as child's age, diagnosis, or prognosis, when explaining their approach. Only two responses (*n* = 2/31) explicitly considered family circumstances as a factor. No clinicians referred to using guidelines, protocols, or shared decision‐making frameworks. Many described the decision as “complex” or “case‐dependent,” suggesting a reliance on individualized approaches. Some clinicians reported deferring decisions to other teams or delaying transitions in the absence of clear pathways.

#### Final comments field (*n* = 33)

Thirty‐three clinicians provided final comments at the end of the survey. The most frequently raised comment was having had minimal personal involvement in a child's tube feeding care (*n* = 11, 33%), with these clinicians citing reasons, including covering others, only brief involvement with tube feeding, or stating tube feeding was not their responsibility. Other recurring concerns included frustration regarding the lack of clarity on tube exit plans and weaning protocols (*n* = 4, 12%), inconsistent approaches to family education and training (*n* = 3, 9%), and passive continuation of tube feeding in the absence of team decisions (*n* = 2, 6%). Several clinicians (*n* = 2, 6%) highlighted the wide range of temporary tube duration from short‐term (24 h) use to months‐long support for children with complex needs, which made some survey questions difficult to answer generically.

### Clinician interviews (qualitative analysis)

**Figure 2:**
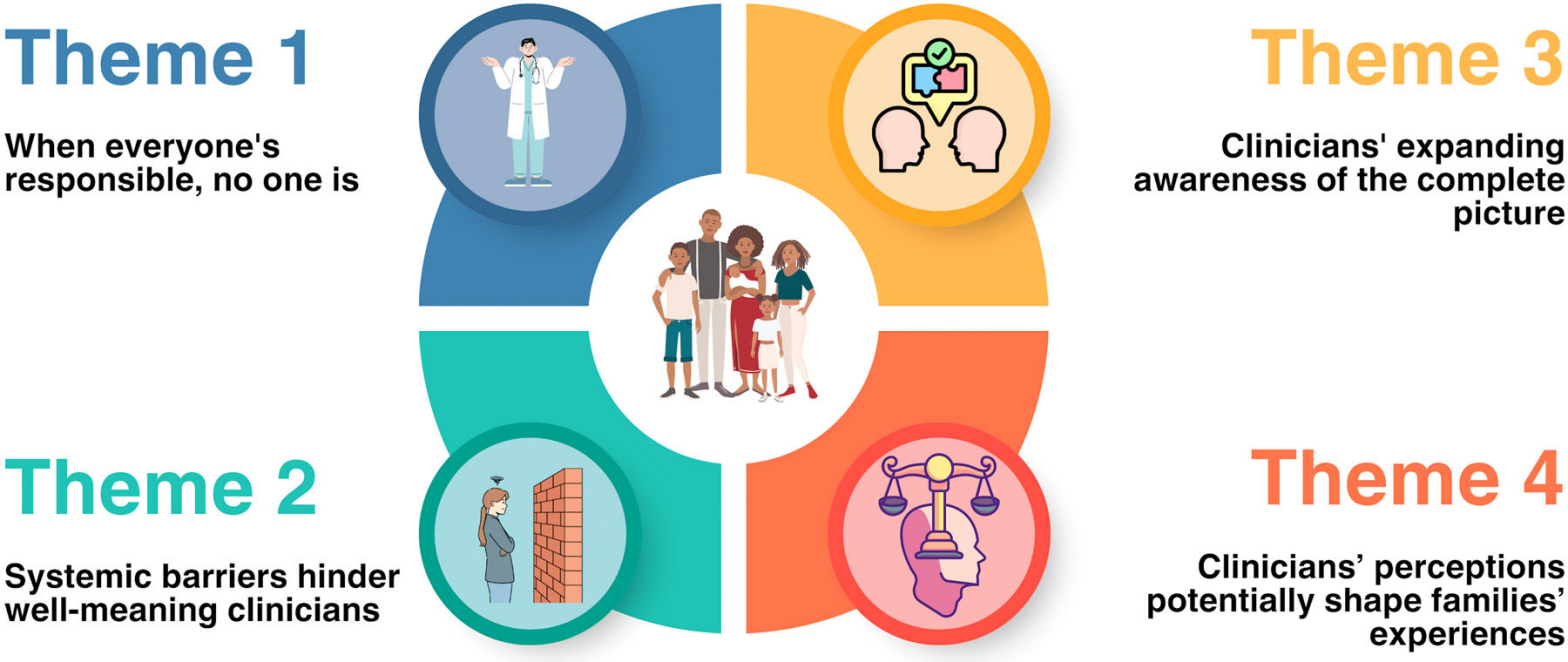
Clinician perspectives on temporary feeding tube management. Themes identified from clinician interviews (*n* = 12) describing role ambiguity, systemic barriers, awareness of family burden, and how clinician perceptions shape family experiences.

Sixty‐four clinicians volunteered and 12 clinicians were selected to participate in semistructured interviews to ensure a range of professions and years of experience were captured. Considerable time elapsed between initial volunteer registration (survey completion was August to December 2023) and interview scheduling (April to November 2024), which reduced the available participant pool. Interview invitations were sent systematically to volunteers across clinical categories to ensure representation from medical practitioners, allied health clinicians, and nurses. When response rates varied across disciplines, selection prioritized diversity in years of experience and clinical roles to capture the widest range of perspectives. This systematic approach ensured the sample reflected varied professional viewpoints rather than predetermined responses. Participants included medical practitioners (*n* = 4), dietitians (*n* = 2), speech pathologists (*n* = 3), and nurses (*n* = 3), with a mean of 14.9 years of pediatric experience (SD = 10.6) and 12.4 years of tube management experience (SD = 9.4). This diverse sample provided rich data and information reflecting a range of clinician perspectives on the management of temporary feeding tubes for children.^46^ Interviews were conducted via Zoom or Microsoft Teams (average duration 48 min, range 18–75 min), recorded with consent, and transcribed verbatim. To maintain anonymity, interview quotes are labeled using unique participant identifiers in the format of (number‐discipline, in which discipline codes include N = nurse, DT = dietitian, SP = speech‐language pathologist, MP = medical practitioner, eg, [01‐N]). Reflexive thematic analysis identified four major themes capturing how clinicians navigate the complex care of children with temporary feeding tubes, from systemic obstacles to evolving awareness of family experiences. Each theme provides in‐depth insight into this clinical environment. A visual summary of themes is provided in Figure 2.

#### Theme 1: When everyone's responsible, no one is

Theme 1 describes how the majority (9/12) of clinicians demonstrated variability in their understanding regarding the specific roles and responsibilities for temporary tube feeding management (see Table 2 for subthemes, categories, and exemplary quotes). Despite the involvement of multiple clinicians, differing perceptions of responsibility meant that clear ownership remained elusive, with 10 clinicians describing role ambiguity, deferral to others or uncertainty about who was responsible for key aspects of tube feeding care. One clinician exemplified this variability in perception, stating “I can't say that I've ever been the one to initially bring up the idea of permanent tube feeding [with a family]… you hope that someone in the medical team at some point has flagged that” [03‐SP], demonstrating uncertainty about both their own role and medical team responsibilities in initiating tube feeding discussions. No clinician described a clear, team‐wide understanding of roles and responsibilities, and instead, most highlighted individual assumptions, professional silos, or uncertainty about what others were doing. Interviews captured that individual clinician philosophies often shaped clinical practice variations in this context. This was described by three clinicians, for instance, a medical practitioner shared their view that “every child should have a general pediatrician who coordinates their care” [07‐MP]. Role delineation was frequently described as unclear by eight of the clinicians. As one clinician reported, “Sometimes if the team aren't sure what's happening, or they're not confident on the plans going home, that can make my job really hard in what I'm recommending to the family… so when do I tell them [family] about the feeding plans, the team come in and say something different” [02‐DT]. Although the rhetoric of collaboration was present, a clear hierarchical structure meant medical teams were viewed as the final decision‐makers, as one medical practitioner stated, “that's the way the medical hierarchy works. The consultant is ultimately directing the care of the patient” [09‐MP]. This perspective was shared by six clinicians, across both medical and allied health roles.

**Table 2:** Theme 1: When everyone is responsible, no one is.

Subtheme	Level	Example quote
Personal philosophy drives practice	Clinical (micro)	“I believe if ultimately like we deem the parent and carer to be able to make like some decisions for their child. Uhm, they have the ultimate right” [04‐MP].
No one person owns the role of assessing family capacity	Clinical (meso)	“I work between four medical teams, I'm guided by them [their decision about a child going home with a feeding tube]” [06‐N]. “[The] senior nurse, the CNC or myself. will be able to say that family are competent” [10‐N]. “I think most units have the nursing team have, you know, their own sort of in‐house checklist [for assessing capacity]” [09‐MP].
Shared care, but medical team as default decision‐makers		“I still think the main person should actually be the general pediatrician” [07‐MP]. “[Medical team] have the final say. Of course, they're kind of the overarching care medical team, their treating team. They certainly rely on allied health and dietetics and speech to, to give that informed decision, but it ultimately comes down to them” [02‐DT]. “So at the end of the day, it does come down to the medical decision. and I think [medical teams have] varying levels of …receptiveness to our recommendations” [03‐SP].
Whose job is it—and who is actually doing it		“all the children …who are going to go home on nasogastric feeding, that education is usually provided by the dietitians” [07‐MP]. “I think the nursing staff and allied health would bear the brunt of that support for families with tube feeds at their …clinic appointments” [08‐MP]. “Our nursing staff often provide that support role as well. They're not, usually doing sort of nursing tasks they're doing, kind of social and wellbeing kind of work as well with families” [01‐DT].

However, despite this ultimate authority, the practical implementation and ongoing management of temporary feeding tubes was often perceived by participants as largely falling to nursing and allied health teams. Perspectives on these responsibilities often differed between clinical roles, culminating in assumptions about who would take ownership of specific tasks. These assumptions were described by four allied health clinicians, and three medical practitioners. One clinician assumed, “I think the nursing staff and allied health would bear the brunt of that support for families with tube feeds” [08‐MP], whereas allied health clinicians often felt more uncertain about initiating discussions on long‐term options: “I think it can always be a little bit tricky as to who I guess, who the responsibility falls on to have that conversation” [11‐SP].

#### Theme 2: Systemic barriers hinder well‐meaning clinicians

Theme 2 revealed a healthcare system in which clinicians felt challenged by systemic barriers across interconnected microlevels, mesolevels, and macrolevels, impacting clinical care for children and their families (see Table 3 for subthemes, categories, and exemplary quotes). These interconnected levels, which will be explored below, are the microlevel (individual clinical practice and direct patient care), the mesolevel (organizational structures and interteam dynamics), and the macrolevel (broader systemic or policy influences). Together, these multilevel constraints created a challenging and often frustrating environment in which clinicians struggled to provide the level of care they believed children with temporary feeding tubes needed.

**Table 3:** Theme 2: Systemic barriers hinder well‐meaning clinicians.

Subtheme	Categories	Level	Example quote
Case‐by‐case care without protocols to guide care	1. Unspoken plans 2. Clinical decisions are case‐by‐case decisions 3. Different opinions about tube duration 4. Tube‐related care is reactive, not proactive	Individual clinical care (micro)	1. “The tube weaning or the exit of the tube is, yeah, it's not often discussed when the tube goes in” [12‐N]. 2. “I guess it's trickier in the populations [of children] where it's not as clear about why they would need long‐term [tube] options” [03‐SP]. 3. “So I just really say, ‘we'll just have to see how it goes and we'll review really frequently.’ And I just focus on the frequent reviews rather than a timeframe that I'll give them for a tube wean” [02‐DT]. 4. “If they're a kid that keeps pulling it [the tube] out on a regular basis or a kid that's quite traumatized for sensory or neurological issues with tolerating having that reinserted …then we would look to move [to a long‐term feeding tube] earlier rather than later” [05‐SP].
Learning by doing		Individual (micro)	“I would say, as dietitians, we're not taught this [education on practical management of children with temporary feeding tubes] well…I've just learned from the years of not being very practical myself, probably” [01‐DT].
Clinicians see the cracks in care	1. Inadequate community follow‐up 2. Communication breakdown and mixed messaging 3. Inconsistent discharge and transition processes 4. Limitations in access and follow‐up 5. Lack of clarity and shared plans	Clinical (meso)	1. “The community is not always set up adequately with the knowledge so then we kind of have people who are kind of just in between in no man's land with no real plan moving forward about what we're doing with the tube” [12‐N]. 2. “If all the stakeholders are not on board with the same plan …so that you don't then end up with a situation where [parents] they're not complying with the [tube feeding] plan or not telling you what they are doing” [05‐SP]. 3. “If it's …the tube's been placed at different [hospital] facilities to their usual facility, how does that get handed over and how does that plan get followed through and making sure that those patients are not getting lost to follow up” [01‐DT]. 4. “I mean, if you're, if you're a family coming in for an appointment once a month, you know a lot could change …and while yeah, we would try to do a phone check in or, you know, families can always call us, again depending on the family that may or may not happen” [03‐SP]. 5. “Sometimes if the [medical] team aren't sure what's happening, or they're not confident on the plans going home, uhm that can make my job really hard in what I'm recommending to the family when I'm doing all of my education” [02‐DT].
Team structure and services influence care		Structure (meso)	“We run into a lot of difficulty with community dietitians managing nasogastric tube feeds and plans just because they don't, they're not as familiar with it as hospital dietitians” [12‐N]. “So teams that aren't familiar with [tube] feeding and it's not their specialty area, they're very reluctant to go down the [tube] feeding pathway” [02‐DT].
Systems constrained by their own design		Structural (macro)	“I believe there is something somewhere [a written resource on tube feeding education for families], just that I'm not aware of the how to find that. But if we have like some resource packs that would be good” [04‐MP]. “sometimes there's quite a bit of pressure to get kids out of hospital…So like from the bed pressure perspective, if the tube is the only thing keeping a child here, the chances are that the medical teams are going to want to push to discharge with the tube” [03‐SP].

*Note*. Numbered categories align directly with their corresponding participant quotes (eg, category 1 with example quote 1), ensuring clear traceability between interpretation and data.

At the microlevel, the absence of clear guidance forced reactive practices. Many clinicians (9/12) reported making ad hoc decisions, especially regarding feeding tube duration, “I just really say, ‘we'll just have to see how it goes and we'll review really frequently. And I just focus on the frequent reviews rather than a timeframe’” [02‐DT]. Additionally, the lack of systemic guidance meant two clinicians relied on experiential learning, “I've just learned from the years of not being very practical [regarding tube feeding plan] myself, probably” [01‐DT]. This reliance on individual experience further contributed to practice variability.

These individual challenges were also seen to stem from mesolevel organizational barriers. Several clinicians described unclear discharge planning, inconsistent follow‐up procedures, long waitlists for long‐term tubes, and fragmented clinical documentation. These issues were raised repeatedly across interviews, all of which were seen to compromise continuity of care and created concerns regarding patient safety. For example, one clinician described the challenge of “finding that burden of what's safe and reasonable versus the actual burden on this family” [02‐DT] when navigating organizational protocols regarding how parents manage tube reinsertions. Team involvement and structure were also reported to play a role in tube management, with four clinicians noting that children often received different care depending on which team was involved: “depends largely on the team, and probably within the teams who you speak to” [03‐SP]. Conversely, three clinicians working within specialist teams in which children often require transitioning to long‐term feeding tubes described more proactive care: “we are usually pretty good, our team, about doing the early referrals [for long‐term feeding tubes]” [01‐DT]. However, half of the clinicians reported challenges navigating the long‐term tube referral pathway:

I would check with the team first to see if clinically that's how they felt… because ultimately it's the medical teams that need to be able to… do the referrals… And it's not as easy as just doing a referral and [having it] accepted… sometimes the surgical, the gastro teams will ask for different tests to be done to ascertain why they need a long‐term tube and things like that. [06‐N]

Perceived disparities between clinical team structures and available services were also seen to directly impact on care delivery. For example, some teams had consistent access to social work support for families, whereas others worked within structures where such support was limited or unavailable, directly affecting the level of psychosocial care clinicians could provide. Six clinicians discussed disparities in access to social work support for families. Some clinicians noted they could “refer on to social work as well for that little bit of emotional support” [11‐SP], whereas others in less‐resourced teams expressed hesitation to even “go down the [tube] feeding pathway” [02‐DT] because of inadequate support structures.

Macro‐level system constraints compounded these issues. Clinicians highlighted challenges, including staff shortages stretching clinical capacity and delaying care, with four clinicians unaware of available temporary tube feeding guidelines to use. Five clinicians identified difficulties in the process of transitioning a child from a temporary feeding tube to a long‐term feeding tube insertion: “it's so hidden to us, the whole ‘who owns the wait list.’ it just feels complicated, even from an insider's perspective” [08‐MP]. Similarly, tube weaning in the hospital was “not nearly intensive enough in order to have a safe outcome” [08‐MP], indicating a potential lack of investment for this important phase of care. One participant discussed how hospital bed pressures were overriding feeding tube management: “there's quite a bit of pressure to get kids out of hospital… if the tube is the only thing keeping a child here, the medical teams are going to want to push to discharge with the tube” [03‐SP]. This reflects how system‐level targets and resource constraints can potentially shape care.

#### Theme 3: Clinicians' expanding awareness of the complete picture

Theme 3 explores the complex nature of clinicians' awareness of the emotional, social, and practical impacts of temporary tube feeding on families. This theme is described in three parts. First, the variation in how clinicians perceive and acknowledge the practical and psychosocial burdens families face is reported. Second, although clinicians demonstrated understanding of families' emotional challenges, these issues were not at the forefront of their minds and were only discussed when directly prompted by interviewers. Finally, this theme examines how this awareness was not static but evolved with clinical experience, leading to more holistic care but also creating inconsistencies in how care is approached for families. See Table 4 for subthemes, categories, and exemplar quotes.

**Table 4:** Theme 3: Clinicians' expanding awareness of the complete picture.

Subtheme	Example quote
Inconsistent recognition of logistical and time burden for parents	“The parents might look terrible in clinic because they're so tired because they've been off doing a very unsustainable [tube feeding] plan overnight” [01‐DT]. “Usually they're [parents] coming in and they're quite flustered, uhm they're not quite remembering the feeding plan, uhm you can just hear them on the phone they're not quite concentrating, uhm they're just exhausted essentially, they're not sleeping” [02‐DT]. “Managing other kids and other people in the household with the tube, making sure …you know like we hear stories all the time of, you know the one‐year‐old's got the tube in and then the three‐year‐old just comes over and like pulls it out …just the logistics of everyday life and having a feeding tube” [12‐N]. “[It can be a] bit daunting for the parents [going home with a tube]” [04‐MP]. “It's just that one extra burden that just sort of tips it over the edge in terms of the actual overall burden of care” [09‐MP].
How clinicians interpret a child's experience is variable	“If they're a kid that keeps pulling it out on a regular basis or a kid that's quite traumatized for sensory or neurological issues with tolerating having that reinserted, it can be quite traumatic for some kids” [05‐SP]. “A baby might have less of an issue with [the feeding tube]” [04‐MP]. “I don't think kids are bothered by it [the tape for the feeding tube]” [06‐N].
Inconsistent awareness of emotional impacts	“If we're finding that a patient is very overwhelmed with the feeding plans …so there's a lot more going on than just the feeding tube at this point usually in my experience anyway” [02‐DT]. “The biggest thing that parents actually don't like with children with nasogastric tube is the fact that everybody stares at them when they go to the supermarket. And that's actually not always well recognized by medical and allied health staff” [07‐MP]. “I think we're generally quite good as well at recognizing that having a child …with the tube is stressful” [11‐SP]. “When a child's sometimes [has] been given this tube, it's a, a grief and loss [for the parents]” [10‐N].
Conversations that do or do not happen	“I think it [telling families about going home with a tube] can just add more stress if you're explicitly saying, ‘this is really overwhelming and it's a lot of work and a lot of effort and, uhm, you know, this will be terrifying’” [02‐DT]. “I think the respiratory physician feels less equipped to say when that end point [of tube feeding] is, so it just keeps going on and on and on” [08‐MP]. “I believe the relationship …the rapport we built …with the family and the child as well. So, if they have like a level of confidence in what our assessment and management plan is, that might be easier to breach the idea [of needing to go home with a tube] and get them on board” [04‐MP].
Experience reshapes practice	“I think we've gotten better since …working with people like [dietitian] at talking about always having a [tube] exit plan” [05‐SP]. “I think probably from working over the years you get more practical and realistic about what [a tube feeding plan] might look like for the family who has, has to do it” [01‐DT].
Approaches that enable effective care	“Yeah, I think the more you do before discharge to know that they're supported, how to practically set themselves up at home …that need to integrate it into their life rather than turn their bedroom into a hospital” [06‐N]. “I think most people [parents] can manage it if they're, they're shown how to do it [use the feeding tube] and they have uhm, you know, an understanding of, of what and why” [09‐MP]. “We've become better at talking about that it's [the feeding tube] a temporary measure, that we would have a tube exit plan …we're never going to send them home and just go, ‘oh well, that seems to be working for you. Off you go’” [05‐SP].

A primary finding was the broad range in how clinicians recognized the daily burdens of tube feeding, with perspectives ranging from acute sensitivity toward practical disruptions to a minimization of the tube's overall impact on the family and child. Although half of the clinicians acknowledged disruptions to daily life, “attendance at daycare can be really tricky…some…have fifty billion hoops that you have to jump through” [11‐SP], a few (2/12) minimized them. For these clinicians, the tube's impact was viewed as secondary, particularly when families were navigating a child with a complex medical condition, with one clinician stating “oh, a tube. That's, you know, the least of your worries“ [03‐SP]. This divergence in perspectives highlights the varied lens through which clinicians understood the daily realities of temporary tube feeding. Similarly, perspectives on children's experiences diverged between several clinicians recognizing direct impacts, “the child can't do swimming lessons anymore” [12‐N], and those assuming minimal effects, with one clinician stating “I don't think kids are bothered by it [the tape]” [06‐N].

Clinicians demonstrated varying levels of awareness regarding families' emotional experiences with temporary feeding tubes. When first discussing the challenges families faced, all 12 clinicians described practical and logistical issues (eg, equipment, training). Subsequent discussion encouraged reflection on potential emotional and psychosocial impacts. During this discussion, all clinicians acknowledged the emotional impacts on parents, such as grief, loss, distress and the exhaustion associated with temporary tube feeding. As one clinician noted, “I guess you know, if the tube comes out and the child has to be held down to have it put back in, and that's stressing everybody, and just like the yucky, emotional stuff…and the dread” [12‐N].

In addition, a few clinicians described softening or delaying emotionally difficult discussions with families: “I think [telling families about going home with a tube] can just add more stress if you're explicitly saying ‘this is really overwhelming…’” [02‐DT]. Others reflected on the importance of relationship‐building to enable such conversations, “I believe the rapport we build with the family… makes it easier to broach the idea [of home tube feeding]” [04‐MP]. Only a few clinicians (2/12) described how their approach to care evolved over time, allowing them to better address both the practical and emotional dimensions of care. This involved becoming more practical and realistic about home management, as one clinician shared, “The more preparation we can do before discharge, recognizing it's not just the feeding tube but how they'll manage at home, sets them up for a better time at home” [06‐N].

#### Theme 4: How clinicians' perceptions potentially shape families' experiences

Theme 4 showed how clinicians shaped families' experiences with temporary feeding tubes, creating structures that both enabled and constrained families' participation because of the inherent power dynamics (see Table 5 for subthemes, categories, and exemplary quotes). Several clinicians spoke about navigating complex decision‐making, acknowledging families' rights to make decisions: “I believe if ultimately … we deem the parent and carer to be able to make … some decisions for their child … they have the ultimate right” [04‐MP] while concurrently framing tube feeding as a medical necessity where “but obviously if a child comes in who's severely malnourished they don't really have choice [regarding a tube insertion]” [07‐MP]. This tension was evident as families often raised tube weaning plans, with one clinician describing how parents typically initiated these discussions by stating: “we don't want this [feeding tube] in at all… we want to get rid of this” [06‐N]. This paradoxical positioning could present an inherent tension in families perceived agency and role in decisions. Clinicians' judgments about individual family capacity were reported to directly determine the nature and intensity of support provided. Six clinicians described how assessments of whether families were in a “fairly stable environment” [07‐MP] or seemed “quite interested and manage [the tube] well” [07‐MP] were seen to be foundational. When families were perceived as capable, support often focused on providing essential knowledge and practical skills, reflecting the belief that “most people [parents] can manage it if they're, they're shown how to do it…and they have…an understanding of…what and why” [09‐MP]. Conversely, if barriers to family learning or management arose, these were often framed as family deficits. Two clinicians described how such assessments typically triggered a shift from enablement to surveillance: “if there's still barriers to teaching them…it usually goes along with a child safety sort of pathway from my experience” [06‐N]. In this context, “child safety” referred to the involvement of child protection services where concerns exist about a family's capacity to manage care safely.

**Table 5:** Theme 4: How clinicians' perceptions potentially shape families experiences.

Subtheme	Categories	Example quote
Relying on parents to raise concerns or figure it out		“Families will bring the question [about a long‐term feeding tube] up to you and kind of go ‘I've done some research like what do you think about this?’” [11‐SP]. “Quite a lot of them [parents] by the time they come and see me have had it for quite a while and they're quite keen to get rid of the nasal tube” [06‐N]. “I've had families that turn internationally to tube weaning programs that they do completely independently of advice locally” [12‐N].
Judging family capacity in unequal contexts	1. Perceived parental competence and readiness 2. Psychological and environmental challenges 3. Monitoring and backup systems 4. Risk and safety concerns	1. “So first of all is parents like or carer's level comfort with handling and managing the NG tube, and if they have the time, capacity and availability to do that as well, cause uhm understandably some parents might have like lots of other commitments or lack of social support that might make it a bit tricky to manage that at home” [04‐MP]. 2. “if they're in a fairly stable environment, most of these kids are good, and most parents are actually quite interested and keen, and they manage [the tube] well for their kids” [07‐MP]. 3. “So …nine times out of ten there'll be some sort of social work involved [with the family]…So, yeah, a lot of them would have some sort of mental health support” [02‐DT].
A tube is a medical call, not a conversation		“Obviously if a child comes in who's severely malnourished, then they don't really have choices [about needing a tube]” [07‐MP]. “Their [tube] feed plan is so precise. If they don't get it or get behind on their feeds, that's going to be a …hospital ICU admission. Possibly death” [01‐DT]. “So if they've demonstrated unsafe swallow will be obviously the main …clinical indicator of feeding the tube” [08‐MP].
Family involvement, but guided by clinicians		“We, we talk a lot about having families present an ideal scenario of what feeding would look like for them and then trying our best to support that, but without promising them something that we can't promise” [05‐SP]. “Of course we need to have parents/carers to be on board with the idea [of their child going home with a feeding tube] as well” [04‐MP]. “The family I think are super important, they should definitely be involved in that decision [of their child going home with a tube]” [11‐SP].
What parents need to succeed	1. Building practical competence and confidence 2. Tailoring education to family learning and context 3. Establishing understanding and rationale 4. Supporting sustainable and real‐life integration	1. “It's just breaking it down to make it so that they can envision how they're going to adapt at home. In their own environment [with their child and the feeding tube]” [06‐N]. 2. “if they need a video of how to do things with the tube, or positioning those sorts of things, if, if it's helpful for them to have video” [05‐SP]. 3. “So we have tried to make it more routine of really reiterating the importance of …using the tube when it's there, it's not there …just for fun” [11‐SP]. 4. “We do take into account all the considerations around what's happening for that family and for that person at that period in time” [01‐DT].
Culture, language, and assumptions about care		“English as a second language is often a barrier …that just kind of immediately adds another level of difficulty for families” [12‐N]. “Like in some culture it might be like, uh not quite accepting towards like having, like an NG support” [04‐MP].

*Note*: Numbered categories align directly with their corresponding participant quotes (eg, category 1 with example quote 1), ensuring clear traceability between interpretation and data.

Furthermore, assumptions about families, particularly regarding culture and language, seemed to influence clinical judgments and interactions. Two clinicians attributed resistance to feeding tubes to cultural preferences, with one explaining that “often culturally and linguistically diverse families will have strong, I guess, cultural attachments to oral feeding. So sometimes… they're the families that are less likely to want to go home with the tube” [03‐SP]. Linguistic differences were described as barriers rather than opportunities for adapted, culturally responsive teaching. Other clinicians reflected on the presence of personal beliefs, stating “you carry personal beliefs whether you realize you do or not” [05‐SP], and expressed a desire for care improvements, such as “if we can get like…cultural support workers…that might be easier in bridging the gap” [04‐MP].

## DISCUSSION

This study provides new insights into how clinicians navigate temporary pediatric feeding tube management in the absence of evidence‐based clinical guidelines, revealing systematic tensions within current healthcare structures that limit clinicians' ability to support families. By integrating quantitative survey data with rich qualitative accounts from multidisciplinary clinicians, this research exposes how key drivers, such as system structures, communication between teams and families, and variability in responsibilities, influence clinicians' abilities to care for families of children with temporary feeding tubes. Through comparing survey responses with interview accounts, this analysis identified three pivotal drivers that present opportunities for improving temporary feeding tube management. These drivers involve structured interprofessional collaboration, congruence between family‐centered intentions and clinical understanding of family experiences, and consideration of both medical stability priorities and psychosocial burden from prolonged temporary tube use.

### The need for structured clinical frameworks

The first main finding reveals how clinicians often needed to navigate temporary tube management in an unstructured format, despite the complexity of this intervention. Survey data demonstrated that clinicians relied heavily on collegial advice for decision‐making regarding temporary feeding tube management. Although collegial consultation represents valuable clinical expertise, this pattern suggests challenges in integrating formal evidence into practice when structured guidance is absent or inconsistently implemented, rather than indicating that evidence does not exist or that clinicians lack skills to access it. Limited guideline awareness is well‐documented in the literature. In previous research, only 27% of multidisciplinary pediatric clinicians were aware of existing tube feeding practice guidelines,^16^ and only 53% of pediatric medical specialists were familiar with the European Society of Paediatric Gastroenterology, Hepatology and Nutrition (ESPGHAN) tube feeding guidelines.^34^ Research demonstrates that when tube feeding protocols are unclear, less experienced clinicians default to seeking advice from senior colleagues, even when their colleagues' practices are variable.^12^ Knowledge gaps and unclear training protocols further contribute to this reliance on informal guidance.^9^ Key barriers to clinical guideline adherence include not only practical constraints, such as time, but also fundamental issues of guideline awareness, familiarity and clinician agreement.^47^ This pattern of findings align with broader healthcare research highlighting how evidence‐based limitations and implementation barriers contribute to practice variations and reliance on institutional knowledge.^11^, ^48^

Clinical practice was also characterized by significant role ambiguity. The infrequent use of formal multidisciplinary meetings may have contributed to the ambiguities observed, since informal discussions may lack the structured documentation, clear decision‐making frameworks, and systematic follow‐up that formal multidisciplinary processes provide. Without standardized meeting structures, decisions depend on clinician availability and individual approaches rather than systematic protocols. This inconsistency was evident in interview accounts that discussed contradictory authority structures. Medical teams were considered to have final hierarchical authority, yet survey responses indicated they were perceived as less frequently involved in decisions than allied health clinicians or parents/caregivers.

Clinicians frequently described nurses as de facto coordinators despite lacking formal authority. Research supports this finding, indicating that nurses are routinely identified as coordinators in tube feeding management^49^ and for children with complex medical conditions.^50^, ^51^ However, this informal assignment of coordination responsibilities can create tension, in which nurses simultaneously report uncertainty about their responsibilities in tube feeding care and feel disempowered in their roles.^52^ Additional research suggests that pediatric nurses depended on dietitians to manage tube feeding plans, reporting limited tube feeding knowledge.^35^ This gap between what nurses are expected to coordinate and what they feel equipped to manage exemplifies how informal role assignments can create coordination challenges when expectations exceed knowledge and expertise.^9^ These role ambiguities reflect broader systemic issues in which clinicians hold different beliefs and overlapping responsibilities, leading to management and coordination challenges.^53^, ^54^

These coordination challenges are likely amplified by the large diversity of children requiring temporary feeding tubes. An audit of 494 children revealed temporary feeding tubes were used across a wide range of medical conditions and indications.^4^ Although children with specific medical conditions have structured protocols, such as preterm infants in NICU settings^30^, ^55^ or children with inflammatory bowel disease requiring standardized feeding timeframes,^56^ this heterogeneity means clinicians frequently encounter unfamiliar combinations of medical complexity and feeding challenges that lack clear management pathways. This population‐based variation in available guidance directly contributes to unclear role delineation, with different clinical teams developing their own approaches of management.^48^

The combination of informal evidence consultation, role ambiguity, and population‐specific guidance gaps creates challenges for consistent clinical decision‐making within the current system structure. Such unclear ownership contributes to reliance on ad hoc approaches, which can increase risk of adverse clinical events.^57^ In line with previous literature, survey responses in this study showed clinicians' need for clearer guidelines, more multidisciplinary input, and training,^5^, ^16^, ^33^, ^58^ indicating that structured collaboration frameworks could address these fragmentation challenges while preserving the valuable teamwork and clinical expertise already demonstrated.

### Disconnects in family‐centered understanding

The findings revealed disconnects between clinicians' family‐centered intentions and their understanding of family experiences. Although survey responses demonstrated that clinicians consistently endorsed family involvement, interview discussions revealed gaps in how families' perspectives, values, and concerns are integrated into clinical practice. This disconnect may limit the family support that clinicians are able to provide. Research has demonstrated that clinicians consider parents' requests regarding their child's temporary feeding tube as their lowest considerations when determining whether a child should transition from a temporary feeding tube to a long‐term feeding tube.^5^ Lively and colleagues found that when parents felt isolated from and disrespected by the treating team managing their child's feeding tube, they disengaged from the health service.^59^

This disconnect reflects the biomedical framework predominant in hospital settings, in which biomedical concerns often overshadow psychosocial considerations.^60^ Clinicians and families prioritize different needs and values regarding feeding tubes.^18^ Although clinicians in this study demonstrated a sophisticated understanding of families' emotional experiences, using nuanced language, including grief, stress, stigma, and social burden to describe family challenges, these impacts were not the first challenges discussed by clinicians. Clinicians first described the medical and physical impacts of temporary tube feeding. Previous research has reported that health structures that implicitly discourage emotional engagement compound this issue, in which emotional support falls outside clinicians' perceived scope even when they understand these impacts.^60^ Such compartmentalization suggests that hospital‐based health models may not adequately support clinicians to integrate biopsychosocial approaches, despite research emphasizing the crucial need to consider families' emotional burden when children require temporary feeding tubes.^61^, ^62^ However, in the current study, only two clinicians described how their care evolved to address both families' practical and emotional needs. This limited reflection suggests few opportunities for structured learning or adaptation, which may constrain clinicians' ability to respond holistically to families over time.

Cultural assumptions further compounded these disconnects, with clinicians attributing tube feeding resistance or management difficulties to cultural factors rather than exploring individual family circumstances. Interview accounts revealing assumptions of cultural and linguistic diversity were framed as barriers to temporary tube feeding acceptance and management rather than factors requiring tailored support approaches. However, research has established that cultural fears and negative attitudes toward temporary tube feeding can be effectively addressed through clinical education, with one study showing significant improvements in caregiver attitudes (from 68% to 100% positive attitudes) and knowledge following culturally appropriate interventions,^63^ whereas other research emphasizes the need for culturally, linguistically, and contextually relevant approaches in tube feeding decisions.^64^ These patterns demonstrate how well‐intentioned clinicians can sometimes overlook the complex emotional and cultural realities families experience with temporary tube feeding, ultimately undermining the therapeutic relationships essential for effective management.

### The contradiction of medical necessity vs psychosocial burden

The findings from this study center on the variability and lack of consensus regarding when to transition from temporary to long‐term feeding tubes, with recommendations ranging from 4 to 18 months. This uncertainty creates tensions between achieving medical stability and acknowledging the considerable burden temporary feeding tubes place on families. Without clear institutional frameworks, clinicians may delay difficult decisions about tube weaning or long‐term tube referrals to avoid potential harm.^60^ Additionally, framing temporary tubes primarily as medical necessities allows biomedical criteria to systematically override psychosocial costs, perpetuating prolonged temporary tube use.^5^, ^65^ This pattern of individualized, variable approaches has been documented repeatedly for over a decade^9^, ^16^, ^34^, ^66^ yet persists clinically. The consequence of these system constraints becomes evident in practice, with children having temporary feeding tubes for prolonged periods without families being involved in decisions about duration, despite their preferences for ongoing discussions about their child's tube's trajectory.^41^, ^67^, ^68^, ^69^ This paradoxical positioning of families as both self‐advocating participants and passive recipients could create considerable confusion and anxiety, leaving families unsure of their agency and role in decisions, thereby potentially impacting their trust, engagement, and preparedness. Most revealing, Theme 4 findings highlighted that parents often initiated tube transition discussions and sought their own solutions, suggesting the need to formally recognize and include families as experts in their own needs and care to enhance clinical decision‐making and care coordination. Parent‐initiated advocacy aligns with broader research findings demonstrating that without transparent discussions with clinicians, many parents navigate tube weaning options independently.^59^

### Clinical implications

This study highlights several clinical implications relevant across diverse healthcare settings. Evidence confirms that implementing standardized and codesigned education protocols for caregivers of children with gastrostomy tubes significantly improved outcomes by reducing clinical complications and unplanned hospital visits,^70^, ^71^ decreasing caregiver burden and anxiety,^70^, ^72^ and increasing knowledge and confidence.^70^, ^71^, ^73^ Although these studies focus on long‐term tubes, the core principles of structured caregiver education, clear pathways, and burden‐aware support apply equally to temporary tube feeding.

To align clinical practice with these findings, clinicians should ensure that the feeding and treatment plans of temporary tube placements actively incorporate family goals and priorities.^19^ Engaging and partnering with families is a crucial component of safe clinical practice, leading to better healthcare outcomes and increasing clinician confidence and satisfaction.^74^

Supporting more consistent approaches to temporary tube feeding clinical practice may require a combination of workforce, communication, and system‐level strategies, such as clearer role delineation, standardized pathways, and specific clinician training.

At the clinical team level, interventions such as clearer role delineation, dedicated coordination roles, and clinician training in communication and family burden assessments could help reduce role ambiguity and promote more consistent, holistic family‐centered care.^13^, ^75^, ^76^ System‐level priorities may include formalizing referral pathways and developing criteria to guide tube transition decisions, potentially reducing unnecessary delays in long‐term tube placements. Implementation processes might benefit from structured tools, such as checklists,^63^, ^77^ decision aids,^5^, ^78^ and digital goal‐setting resources,^79^ supported by routine clinical audits and feedback monitoring. Future efforts to develop, implement, and evaluate such interventions could draw on established implementation science frameworks.

### Limitations

Several limitations warrant consideration. Although the 54% response rate compares favorably with clinician surveys,^80^ we could not characterize nonresponders; therefore, nonresponse bias cannot be excluded and findings may not be generalizable to all clinicians.^81^ The survey's multiple‐choice format may have influenced responses by making expected answers apparent, potentially limiting nuance and inflating reported practices through social desirability bias, despite this being the preferred choice of clinicians during pilot testing. When clinicians could see response options such as “always involve families,” they may have selected what appeared professionally appropriate rather than their actual clinical practice.^82^, ^83^ The term “hospital guidelines/policies” was not defined in the survey, limiting our ability to identify which specific documents informed clinicians' responses. Also, it is recognized that not all children requiring temporary feeding tubes will require multidisciplinary team input, particularly those receiving short‐term hospital‐based tube feeding for acute conditions, so these results should be interpreted accordingly. Interviews were conducted with clinicians at a single site, and therefore, it is acknowledged that the experiences of clinicians working at differing sites may provide further insights into the drivers of temporary tube feeding in clinical care. In addition, clinicians' years of experience were not collected in the survey, limiting the ability to explore whether experience influenced responses or contributed to differences in reported clinical practices.

## CONCLUSION

This study addresses an important research gap by describing clinician perspectives on the tensions between clinical priorities and family needs and identifying how clinicians navigate temporary tube feeding management. Although clinicians demonstrated an awareness of family challenges, the findings that psychosocial impacts were only discussed when prompted suggests current healthcare structures may not adequately prioritize family‐centered approaches. These findings indicate that developing standardized protocols and evidence‐base guidelines could directly address the variability identified in this research. Supporting more effective clinical practice likely requires interventions such as evidence‐based tools (eg, checklists, decision aids, goal‐setting resources), communication training, defined roles, and coordinated care pathways that are embedded within systemic standards of care. Future research should prioritize codesigned interventions that support families with temporary tube feeding management in both hospital and home settings, including developing guidance for clinicians on how to have difficult conversations with families. Further work is needed to explore clinical decision‐making approaches that align with family goals during temporary tube feeding care. Additionally, larger studies examining discipline‐specific and consumer perspectives could provide valuable insights for developing targeted training and education.

## AUTHOR CONTRIBUTIONS

Claire Reilly, Rebecca Packer, Jeanne Marshall, and Nikhil Thapar contributed to the conceptualization and design of the study; Claire Reilly contributed to data collection, data curation, and formal analysis, with Jasmine Foley contributing to data analysis; Claire Reilly prepared the original draft; Claire Reilly, Jasmine Foley, Rebecca Packer, Jeanne Marshall, and Nikhil Thapar critically revised the manuscript; and all authors agree to be fully accountable for ensuring the integrity and accuracy of the work and have read and approved the final manuscript.

## CONFLICT OF INTEREST STATEMENT

None declared.

## Supporting information

Supplementary Material.
