# Supplementary material for: Clinician insights into pediatric temporary feeding tube management: Unseen barriers, unclear roles revealed from a prospective mixed methods study
Source: Nutr Clin Pract. 2026 Feb 11;41(4):1205–23. doi: 10.1002/ncp.70093 (PMC13418947; doi:10.1002/ncp.70093)
Supplement: Supplementary file 1 — Supplementary Material. [file NCP-41-1205-s001.pdf]

File S1. Clinician Survey Questions

Please answer the following questions related to the **patient that you were contacted about** with a temporary feeding tube

- ○ Yes, I remember this patient
- ○ No, I don't remember this patient

Which statement do you agree with relating to this child's temporary feeding tube?

- ○ I anticipate that this child will need their feeding tube for < 3 months
- ○ I anticipate that this child will need their feeding tube for 4 - 6 months
- ○ I anticipate that this child will need their feeding tube for 7 - 12 months
- ○ Unsure

I have discussed the **duration** of the feeding tube with the parents +/- child

- ○ Yes
- ○ No
- ○ Unsure

There is a **tube exit plan** in place for this child

- ○ Yes
- ○ No
- ○ Unsure

I have provided **written information** about the feeding tube to the parents +/- child (e.g. a feeding plan, information on tube feeding)

- ○ Yes
- ○ No
- ○ Unsure

<u>The following questions are related to temporary tube feeding in general.</u>

Thinking about your clinical practice in the last 12 months managing children with temporary feeding tubes, what **information** helps your decision-making and **how often** do you use it?

	Never	Sometimes	About half the time	Most of the time	Always
Hospital guidelines/ policies	○	○	○	○	○
Online clinical database (e.g. UpToDate)	○	○	○	○	○
Online search (e.g., Royal Children's Hospital Melbourne website)	○	○	○	○	○
The child's treating team	○	○	○	○	○
Dietitians	○	○	○	○	○
Research journal articles	○	○	○	○	○
Workshops/conferences	○	○	○	○	○
Other	○	○	○	○	○

Thinking about your clinical practice in the last 12 months managing children with temporary feeding tubes, **how** do you typically make decisions about a child's temporary feeding tube?

	Never	Sometimes	About half the time	Most of the time	Always
Discuss with the child's treating team	○	○	○	○	○
Discuss with allied health (e.g., speech pathology, dietitian, occupational therapist)	○	○	○	○	○
Discuss with the family	○	○	○	○	○
Have a multidisciplinary meeting	○	○	○	○	○
Other	○	○	○	○	○

Thinking about your clinical practice in the last 12 months managing children with temporary feeding tubes, **who** do you think should be involved in the decision making of the child's temporary feeding tube?

	Strongly disagree	Somewhat disagree	Neither agree nor disagree	Somewhat agree	Strongly agree
Child's treating team	○	○	○	○	○
Dietitian	○	○	○	○	○
Parents / caregivers	○	○	○	○	○
Feeding therapists (e.g., speech pathologist, occupational therapist)	○	○	○	○	○
Other	○	○	○	○	○

When would you think that a child should transition from a temporary feeding tube to a longer-term feeding tube (e.g., a gastrostomy tube)?

○ 1 - 3 months

○ 4 - 6 months

○ 7 - 12 months

○ 13 - 18 months

○ Other

<u>The following questions are related to **this research study.**</u>

The aim of this study is to understand the decision-making and management of children requiring temporary feeding tubes. How important do you think this study is to the clinical practice for children with temporary feeding tubes?

○ Not at all important

○ Slightly important

○ Moderately important

- ○ Very important
- ○ Extremely important

Would you be happy to be contacted about future research?

- ○ Yes
- ○ No

Would you be willing to participate in an interview (lasting 45 - 60mins) discussing the management of temporary feeding tubes in children?

- ○ Yes - please share your preferred email address to be contacted on:
- ○ No

Do you have any final comments you would like to add?

File S1. Clinician Interview Guide

What is your job title?

How many years have you been working in paediatrics?

How many years have you been managing children with temporary feeding tubes (e.g., nasogastric tubes)?

---

1. How do you approach talking to parents/children about why they need to go home with a temporary feeding tube? Prompt: Are there any resources you might use/ provide during this conversation?

2. How do you educate parents (and children) going home with the temporary feeding tube?

3. What factors influence your decision-making when it comes to discharging a child home with a temporary feeding tube?

4. Who do you believe should be involved in the decision-making process regarding a child's temporary feeding tube? (Prompt: and why?)

5. Who do you believe should hold primary/ultimate responsibility for a child discharged home with a temporary tube? (Prompt: and why?)

6. What factors do you take into account when determining the duration of time a child needs a temporary feeding tube?

7. How do you determine when/if a child should transition from a temporary feeding tube to a longer term feeding tube (e.g., a gastrostomy tube)?
   Prompts:
   Would you have this conversation with the parent/child?
   If yes, are there any resources you might use/ provide during this conversation?

8. What do you perceive as the main challenges faced by children/parents regarding the temporary feeding tube? Prompt: how do you address these challenges?

9. Can you describe a situation where you encountered challenges supporting a child with a temporary feeding tube?

10. In your experience, what factors play a role in determining how a child and their family manages with a temporary feeding tube at home?

11. How do you support children and families managing with a temporary feeding tube at home?

12. Can you describe any insights you've gained about what parents/children consider significant about having a temporary feeding tube?

13. Possible extra question: What would be your recommendation for change regarding the management of children with temporary feeding tubes?
